# Pregnancy, Childcare, and Pesticides: Understanding the Health Risks of Women and Children in Small-Scale Horticulture in Tanzania

**DOI:** 10.1177/10497323251342176

**Published:** 2025-06-13

**Authors:** William Nelson Mwakalasya, Ana Lorena Ruano, Simon Henry Mamuya, Bente Elisabeth Moen, Aiwerasia Vera Ngowi

**Affiliations:** 1 Department of Environmental and Occupational Health, School of Public Health and Social Sciences, 92976 Muhimbili University of Health and Allied Sciences, Dar es Salaam, Tanzania; 2 Department of Global Public Health and Primary Care, Centre for International Health, 1658 University of Bergen, Bergen, Norway

**Keywords:** horticulture, pesticides, pregnancy, women’s health

## Abstract

Women play a significant role in agriculture worldwide, especially in sub-Saharan Africa, where they form a large share of the workforce in horticulture. Horticulture work may expose women to pesticides during pregnancy and childcare, posing serious health risks to women and children. This study explores the experiences of women working in small-scale horticulture in Tanzania during pregnancy and childcare, to understand exposure risks and suggest safety-enhancing interventions. Using the theory of planned behavior framework, five focus group discussions (FGDs) were conducted with women aged from early 20s to mid-40s. The inductive thematic analysis was used for the analysis of the FGDs. We found that gendered roles in horticulture theoretically assign pesticide spraying to men, with women typically handling lighter tasks. However, when men are unavailable, women, even when pregnant, take on all duties, including pesticide spraying. Financial pressures force many women to work during pregnancy and postpartum, sometimes bringing infants into the horticulture environments. Despite some protective efforts reported by women, inadequate safety practices persist due to a lack of formal training on pesticide use. Therefore, traditional gender roles, poverty, and pesticide exposure intersect to create an occupational health challenge for women in small-scale horticulture. The absence of women-centered training on pesticide safety increases health risks both for women and their children. This study recommends gender-sensitive health training, improved access to protective equipment, and economic empowerment for women to help reduce these risks.

## Introduction

Women are integral to agriculture and rural economies worldwide. They constitute 28.5% of the global agricultural workforce (FAO, 2024). Their participation varies significantly across regions, ranging from 66% in sub-Saharan Africa to 71% in Southern Asia (FAO, 2023). In sub-Saharan Africa, cultural norms have traditionally supported female engagement in agriculture, leading to high agricultural participation rates (UNDESA, 2000). Thus, women are central to the economic growth of rural areas in this region.

The emergence of modern agricultural practices has created unique opportunities that have increasingly drawn women into the horticulture sector, where women and youth constitute 65%–70% of the labor force (URT, 2021). Horticulture is defined by the US Department of Agriculture as “a branch of agriculture concerned with growing plants that are used by people for food, for medicinal purposes, and for aesthetic gratification” (USDA, 2024). In several low and middle-income countries (LMICs), such as Iran, Madagascar, Tanzania, and Afghanistan, horticultural crops are often referred to as “women’s crops” due to their high involvement in this field (USAID, 2005).

Horticulture farming involves the intensive use of pesticides, as farmers often rely on them as the primary solution for pest and disease control. Human exposure to these pesticides can result in serious health effects. Women, in particular, are more vulnerable than men due to higher body fat levels and hormonal changes during pregnancy, lactation, and menopause, making them more likely to absorb pesticides and suffer harmful consequences (Atinkut Asmare et al., 2022). In Tanzania, pregnancy complications are frequently reported among women working in horticulture (Mrema et al., 2017). Pesticide exposure during pregnancy is especially concerning, as some chemicals can cross the maternal-placental barrier (Mathiesen et al., 2020), potentially impairing fetal growth and neurodevelopment in children (Buralli et al., 2023; Chilipweli et al., 2021). Consequently, children born to mothers working in horticulture may have a higher risk of adverse health outcomes.

Women, especially in horticulture, are exposed to harmful pesticides due to their vulnerable positions in the workforce. They are often at a higher risk of exposure due to their lower level of pesticide use safety and awareness (Atreya, 2007), the lack of the means to protect themselves, and their exposure risks being normally underestimated (London et al., 2002). These are exacerbated by the lack of attention to women’s unique experiences with pesticide exposure. This study explores women’s experiences working in small-scale horticulture during pregnancy, newborn care, and caring for preschool children who often accompany them to the farms. We uncover insights into pesticide exposure by examining how women navigate their roles in agricultural settings and their awareness of potential health risks. The findings from this study inform the development of targeted interventions that can protect women and their offspring by reducing the toxic impact of pesticides, ultimately fostering safer working environments for expectant mothers in agricultural communities.

## Methodology

### Theoretical Framework

This study utilizes the theory of planned behavior (TPB) proposed by Ajzen (1991) as a comprehensive framework to guide data collection, analysis, and interpretation. The TPB is particularly relevant in this context because exposure to pesticides is significantly influenced by human behavior and activities. In this study, TPB is employed to understand how women’s roles in small-scale horticulture farms influence their behavior toward their body contact with pesticide during pregnancy and newborn care.

According to TPB, an individual’s behavior is shaped by three main factors: one’s attitudes, subjective norms, and perceived behavioral control (Figure 1). The first element, attitude, involves women’s personal evaluations and beliefs about avoiding activities which predispose them to pesticide exposure during pregnancy and newborn care. Subjective norms are the perceived social pressures that the women feel from their family members and fellow farmers regarding engaging in activities that may expose them and their children to pesticides. Perceived behavioral control examines the women’s perceived ease or difficulty in adopting protective measures against pesticide exposure.

**Figure 1.**
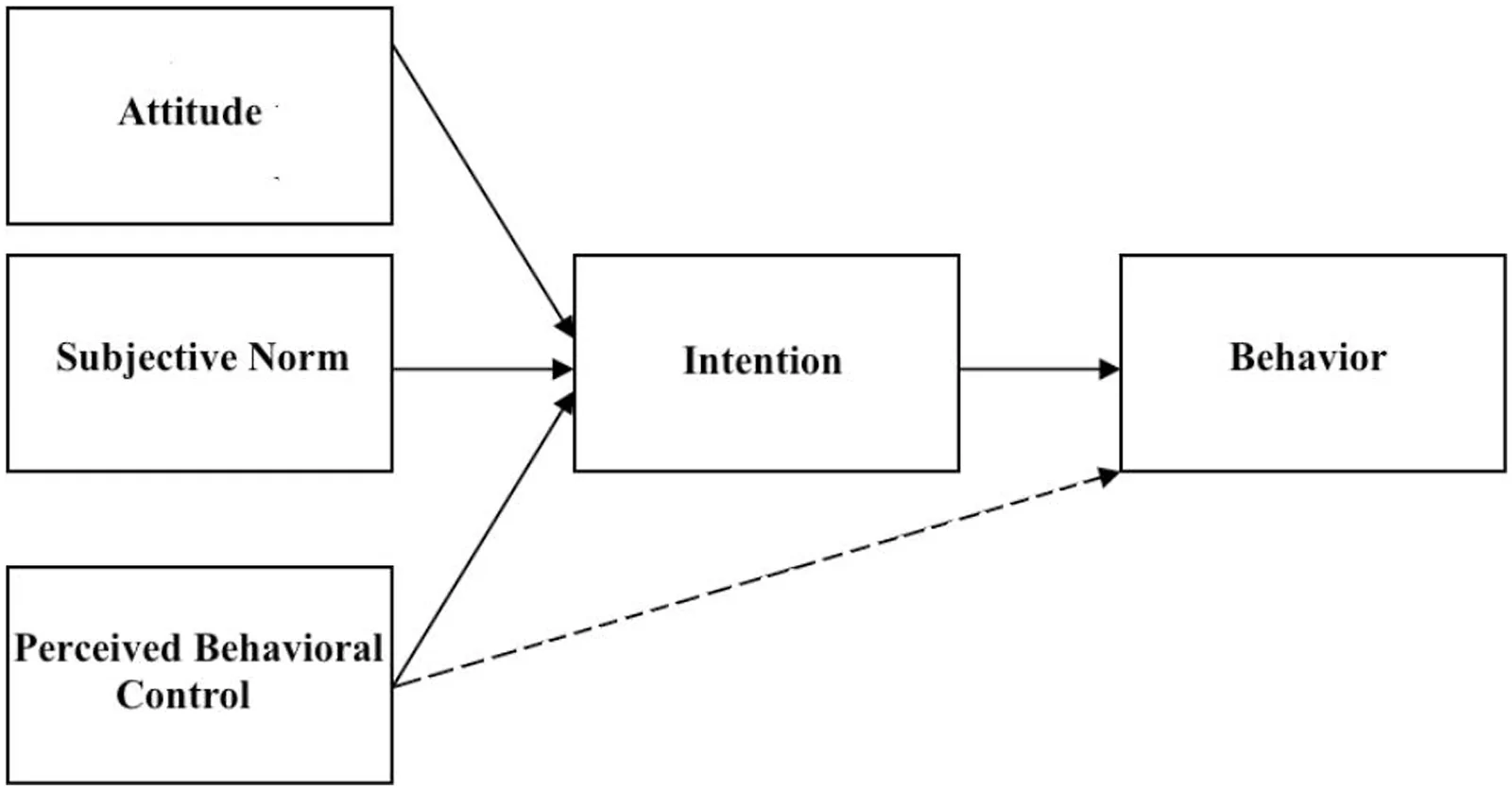
Theory of planned behavior (Ajzen, 1991).

### Study Design

This study employed a descriptive qualitative design by exploring participants’ lived experiences in their natural agriculture setting. This design was chosen because it provides a detailed and straightforward summary of participants’ experiences while maintaining close alignment with their own language and perspectives.

### Study Context

Data for this study were collected from women working in small-scale horticulture farms across one southern region (named A) and two eastern regions (named B and C) in Tanzania, where women constitute a majority of the labor force in agriculture (Leavens, 2011). These regions were purposefully selected because they provide a representative sample of the diverse agricultural practices and cultural contexts within Tanzania, allowing for a comprehensive understanding of women’s roles and behaviors related to pesticide use in small-scale horticulture. Participants from region A were selected from communities where commercial paddy farming is predominant. Utilizing water from irrigation schemes, some families in region A practice both subsistence horticulture farming and commercial paddy farming. Participants from regions B and C were selected from areas where most residents engage in subsistence farming using free-flowing water streams for irrigation, occasionally selling surplus crops in nearby markets.

### Data Collection

In January 2023, five focus group discussions (FGDs) were conducted with a total of 46 participants, who were purposefully selected through face-to-face recruitment facilitated by local farm leaders. All recruited participants provided their consent to participate, and no dropouts were recorded. These FGDs utilized open-ended question guides that were initially prepared in English and subsequently translated into Swahili, the participants’ and moderator’s local language. The participants were women of reproductive age working in horticulture, each with at least one child aged between 4 and 6 years who was born and raised within the same community. These women had also been employed in horticulture before conceiving the child and continued working for some time during their pregnancy. Each FGD had 6 to 12 participants, ranging in age from their early 20s to mid-40s (Table 1). Using the pilot tested interview guide, the discussions were moderated by a research assistant, while the first author documented observations through field notes. All FGDs were tape-recorded and lasted approximately 50–60 minutes.

**Table 1.** Description of Individual Focus Group Composition in Three Regions (Study Areas) in Tanzania.

FGD	Region	Age Range (Years)	Number of Participants
FGD 1	A	20–43	8
FGD 2	A	27–44	6
FGD 3	B	24–46	12
FGD 4	C	21–41	9
FGD 5	C	24–43	8

As urban-based researchers with educational privilege and no direct experience in horticulture, we recognized that our outsider status might affect our interactions with the women and our understanding of their experiences. We were therefore committed to being reflective throughout the research process, addressing potential biases, and prioritizing the participants’ voices in our analysis. During the discussions, the moderator used neutral and open-ended questions to avoid leading participants toward specific responses and remain open to unexpected findings and ensured that conclusions were based on the participants’ voices rather than preconceived notions.

Data saturation was determined through an iterative process during the analysis of the FGDs. After each FGD, the research team conducted a preliminary analysis to identify emerging themes. By the fifth FGD, no new themes or insights were emerging, suggesting that data saturation had been reached. Consensus on data saturation was reached through team discussions, ensuring that the gathered data adequately met the research objectives.

### Data Analysis

All discussions were transcribed verbatim. Data were analyzed using inductive thematic analysis, which is ideal for identifying recurring themes in qualitative data and is suitable for the analysis of FGD-generated data (Braun & Clarke, 2006). Preliminary analysis was conducted after each FGD to get a general impression of the results, which allowed for early identification of areas that needed additional clarifications from the study participants. Researchers conducted the thematic analysis in its original language (Swahili) to maintain trustworthiness and credibility of the findings, which could have been lost by initial, inaccurate translation (Van Nes et al., 2010). Translation into English commenced after themes were generated and for extraction of quotations.

To achieve familiarization with the data, careful reading of the manuscripts was carried out by WNM. Open and axial coding was carried out to capture the complexity of the data collected. By doing iterative coding in this way, we were able to gather understanding around our specific aims while also capturing emerging issues not previously considered by the team. The codes were then clustered into categories. The categories were then examined to identify overarching themes that reflect the core messages of the data. Once the themes were generated, they were narrated to provide a detailed description and interpretation. To illustrate the themes and provide evidence for the interpretations, representative quotes from the participants were selected.

### Ethical Considerations

Before commencing the study, ethical clearance was obtained. At the study sites, permission to visit the wards was sought from the District Executive Directors. During the FGDs, care was taken to ensure that participants fully understood the study’s purpose and provided informed consent. De-identification and confidentiality were strictly maintained to protect participants’ identities.

## Findings

### Theme 1: Gendered Roles in Horticulture Work

The participants in this study expressed that, in rural households, where agriculture is a way of life, the division of labor between men and women reflects traditional practices and practical considerations. Tasks are allocated either to men or women based on their nature and perceived level of difficulty, though sometimes tasks are shared. According to the women in our study, tasks that are considered to belong to men are those which are physically demanding or potentially hazardous such as farm preparation and pesticide spraying. This division allows women to focus on tasks that are perceived as easier, such as planting, pruning, and weeding. However, this division is not strictly adhered to in all households. In some, men may handle non-horticulture crops like paddy and maize, leaving horticulture to their wives only. However, pesticide spraying remains predominantly a male task.

This gendered division is true even in families where both husband and wife work together on all aspects of the farm. Reportedly, there is a clear demarcation when it comes to spraying pesticides: women should then assist with other tasks while their husbands handle this responsibility. In instances where husbands are absent, women find themselves taking on all farm duties, including pesticide spraying. They may occasionally hire men for this, especially when dealing with pesticides. Unmarried women facing similar challenges must often manage to handle all the tasks, including spraying. This might be outsourced to hired workers, but financial constraints sometimes make it difficult to afford hiring workers, forcing them to take on all responsibilities, including pesticide spraying.

We usually divide the tasks based on how we live in our families. Depending on the circumstances, there are certain tasks that women cannot do or certain tasks that men cannot do, and we help each other accordingly. Sometimes there are situations where women are not supposed to handle pesticides, but if it becomes necessary and there is no one else available, they might have to do it to prevent the crops from being damaged. But most of the time, it is the men who handle such tasks. (R2, 30 years old, married, secondary education, from region A)

The consensus among our participants was that the journey of pregnancy does not always signal a pause in work for women. Instead, there is a nuanced adjustment in their roles and tasks. Women typically continue their farm duties, albeit with some modifications. The extent of task reduction during pregnancy depends on individual health and comfort levels, but also on finances. Those facing complications in the first trimester often see a significant reduction in farm tasks during this critical period. However, for those who feel strong, or without a choice, they persist with routine tasks until the later stages of pregnancy or until they experience physical limitations. The help they receive is often dependent on the availability of external support, such as having a partner or the means to hire assistance. Not all of them can afford to take a smaller role, which means they sometimes engage in dangerous tasks like pesticide spray during pregnancy. In some cases, women work until the day before giving birth.

Most of the time, because there are many responsibilities at the farm, you find that there is watering and weeding to be done. So, the man has the responsibility of carrying the pump and spraying the pesticides, while the woman has other tasks in the field. We share the work. The mother cannot wait for the father to finish spraying pesticides to start watering the plants because that would waste a lot of time or lead to crop damage. So, we both participate. Even when pregnant, you have to get up and work. You can’t leave it all to the father because you would waste a lot of time or let the crops get damaged. Therefore, we women are involved in tasks such as planting and even when pregnant, we have to do such work. We can’t leave it all to the father. (R8, 46 years, married, primary education, from region B)

Some women in our study reported helping with mixing pesticides during pregnancy before asking men to do the spray work. This is related to not being able to carry a 20-liter back spray pump and spray pesticides, as it is too heavy for them. However, for women without other options, the burden falls on them and they have to mix and spray themselves. Spraying pesticides is reportedly accompanied by symptoms like headache, nausea, and shortness of breath from the acute exposure to pesticides. Rather than halting work entirely, pregnant women navigate these discomforts by taking intermittent breaks until symptoms subside. This cycle continues during the entire duration of pregnancy.

### Theme 2: We Work When Pregnant or With a Young Baby Because We Are Poor

During the pregnancy period, women should get enough time for resting and sleeping, but this is not the case for women working in small-scale horticulture farms. According to participants in this study, it is the wish of every woman to enjoy a good rest during pregnancy and postpartum period, but they are denied this right because their family depends on the income from horticulture. This is true even if it is not their wish to continue working. This is driven by their financial constraints. Married women will, at least, have assistance from their husbands. The rest will have to keep the farm work going despite the pregnancy and its complications.

It depends on the individual’s life circumstances. You might find someone who gives birth and after two weeks, we meet them in the farm because the home situation is not good. But there are others who give birth and may take several months to return. She stays at home for about three months because sometimes the back pain bothers her, and she may be delayed in returning to farm work. But if we consider the economic situation, three months is not feasible. People think that once the umbilical cord is dropped, they should continue fighting and working. (R1, 37 years, single, primary education, from region A)

These financial constraints mean that a women will not have enough time to heal and nurse their newborns and instead must return to the farms before their bodies are ready and while babies are still young. This has led to the feeling that if it was not for their poverty, they could have enjoyed a protected time for caring for their infants instead of bringing them to dangerous field environments.

After a standard vaginal delivery at a healthcare facility, it is necessary for postpartum mothers to be monitored for at least 24 hours. Following discharge from the healthcare facility after a normal delivery, the experiences of women involved in small-scale horticulture work varied regarding how long it took for them to recover and care for their newborns. Some participants mentioned that there is no set number of days; rather, it depends on how they feel. They resume their farm duties as soon as they feel sufficiently strong. Although it typically takes about a month for many women, some begin with simple farm tasks after about a week and gradually increase their workload as they regain strength. Conversely, others wait until nearly three months, following the advice given by nurses during antenatal care visits. Women who undergo cesarean delivery have a different experience, as they require more time before returning to farm duties, typically between six to even eight months.

It depends on your health, just like it does on mine. If I’m in good health after giving birth and my baby is healthy too, I can spend my forty days and then resume work as usual, considering the situation of life. If you see someone who has stayed for three or four months, it means they are fit and doing well in their life. Now, considering my difficult situation, I’ll have to wait for just the forty days, and I will pray to God that I return to work after forty days to continue farming. (R7, 36 years, married, primary education, from region C)

During the period of returning to the work, the child is still breastfeeding. This makes it difficult to leave the child behind when going to the farm, except for very short trips. In such cases, the child may be entrusted to a nearby relative or trusted neighbor, reportedly for no longer than 2–3 hours. These trips are those involving activities like harvesting for a daily sale or pesticide spraying. Otherwise, for longer trips, the child accompanies the mother to the farm.

At the farm, mothers must strike a balance between work and childcare. Participants in our study reported that some tasks, like weeding, watering, and pruning, can be managed while carrying the child on their back. When dealing with tasks that they cannot manage to do while carrying a child, many women reported to normally seek out shaded spots on the farm or nearby to lay the child down. This was commonly reported to happen on pesticide spray days, regardless of whether it is the mother or another individual performing the spraying. Families with older non-school-going children may bring them along to entertain or supervise the younger child while the mother works.

### Theme 3: Our Children Are Not Safe From the Effects of Pesticides

Depending on the type of pesticide, direct contact can lead to immediate side effects such as skin irritation, headaches, and shortness of breath. In response to these risks, participants reported employing various measures to protect themselves from accidental exposure. Some women mentioned wearing masks or cloth coverings over their mouths and noses while mixing and spraying pesticides, citing the offensive odor of the chemicals as the reason for covering their noses. Additionally, they sometimes wore gumboots during spraying to minimize skin contact.

When I go to the farm and need to spray the pesticide, either my husband or I wear gloves and a mask. I personally wear a headscarf and cover my mouth with a piece of plastic or cloth so that the pesticide doesn’t enter my body through the mouth. Once I have those items on, if we don’t have gloves or plastic, I cover my hands and mix the pesticide. After mixing it, I carry the container on my back and spray it while wearing the headscarf and covering my mouth, even using a cloth to cover it if I don’t have anything else, just to make sure the pesticide doesn’t touch my skin. Once I have all those items on, I enter the farm and spray the pesticide. (R3, 35 years, married, primary education, from region B)

While doing their farm tasks, including spraying pesticides, mothers must also tend to their children. Discussions with participants highlighted measures taken by postpartum mothers to protect their newborns from pesticide exposure. If a child needs attention while the mother is spraying, many women reported only washing their hands before handling the child, though hand washing was not consistently practiced and depended on the urgency of the child’s needs. After completing farm work, women reported changing clothes and showering before holding their children again for the journey home.

In general, in our agricultural environment, it’s a challenge. We can’t say that we seriously wash our hands because we do not go to the farm with soap, only few might. It’s only by God’s protection. Because you find that someone sprays pesticides today, and the day after tomorrow, they are harvesting tomatoes. Those tomatoes we harvest mostly have pesticides, and you work with the child around. When the child cries, you quickly rush over to pick them up and breastfeed them or give them food. There is no soap or anything; it’s only God who protects. (R8, 43 years, married, primary education, from region A)

Participants expressed concerns about breastfeeding infants potentially ingesting pesticides from exposed mothers. Consequently, they adopted protective measures to minimize pesticide transfer. At the end of their work shifts, some women reported drinking milk or plenty of water, believing that this practice diluted any toxins in their bodies, thus reducing the dose transferred to their breastfeeding children.

When you purchase the pesticides, you have to ask for guidance from the shopkeeper on how to use it. Sometimes we get informed by experienced users who tell us which pesticides requires protection because they experienced the effects before. On the day of spraying, they advise us to drink plenty of water or milk to reduce the impact of the toxins. (R4, 44 years, married, primary education, from region A)

Children may sometimes become an integral part of unsafe farm activites, for instance when a mother or someone else is spraying pesticides while the child is at the farm. However, to minimize their direct exposure to pesticides, some mothers in our study mentioned protecting them by covering them with an extra layer of clothing during pesticide spraying.

In small-scale horticulture, pesticides are often mixed using irrigation water streams that pass near the farms. After using pesticides, participants reported leaving containers anywhere because there’s no designated disposal area. As a result, older children on the farms may access these empty pesticide containers and use them as toys. Some participants recounted instances where children used these containers to fetch water from irrigation streams and even drank from them. Additionally, children’s play isn’t limited to specific farms. They sometimes play in recently sprayed fields, where they may pick ripe vegetables like tomatoes to eat them. Consumption of sprayed vegetables also occurs at the family level, with participants reporting the use of vegetables sprayed a day or two years ago for family meals.

Our children are not safe due to the conditions in the farm. For example, you might find a farmer mixing pesticides, then leaving the container right there. When a child comes, they pick up the container and start playing with it. Sometimes we see them fetching water from the channels using containers to drink. They just play around, unaware that there is poison inside. They think it’s normal. (R8, 43 years, widow, primary education, from region C)

### Theme 4: We Need Training on Pesticide Use and Their Side Effects

Awareness of, and adherence to, safety protocols during pesticide handling is important for the health and safety of agricultural workers. These objectives are best achieved by providing farmers with adequate information concerning pesticides and their health effects. However, participants in our study reported a lack of training in this regard, particularly regarding the health impacts of pesticides. Many women stated that they had never received any training related to their farm work. Instead, they relied on guidance from men who had more experience with pesticide use or from the shopkeepers where they purchased the agrochemicals.

Our suggestion, as small-scale vegetable farmers, is that we request the government to assist us by providing seminars or education on the proper use of pesticides. This will help us use pesticides correctly and protect our children and other children who are found in the farm environment. There are children who come to pick vegetables in the farm; if they find a spilled bottle, they might pick it up and use it. My child might know that the bottle contains poison, but the child who comes to pick vegetables may not be aware of the harm it can cause. Therefore, we request the government to consider providing seminars for us small-scale farmers so that we can protect our children and even other children who visit our farms. (R8, 43 years, widow, primary education, from region C)

Literate farmers had some advantages, as they could follow the instructions provided on pesticide packaging labels and leaflets, using them as their primary source of information. These instructions primarily guided them on how to properly mix the pesticides before application. Regarding the choice of protective clothing, most participants relied on their own experiences of acute health effects following pesticide use. Based on their previous symptoms, they would cover the affected body parts during subsequent applications to avoid experiencing the same discomfort again. As a result of these findings, participants emphasized the necessity of training on safe pesticide handling practices.

## Discussion

Aligned with the TPB framework (Figure 2), this study reveals that gendered roles persist in horticultural work within rural households, particularly concerning pesticide use, which remains a, theoretically, male-dominated task. This also showed that women are generally thought of as only engaging in less physically demanding activities like planting and weeding, while men handle pesticide spraying due to the perceived hazards. However, when men are unavailable, women, even pregnant ones, perform all farm duties, including spraying. Financial constraints compel many women to continue working during pregnancy and the postpartum period, sometimes taking their infants to hazardous environments. Despite efforts to protect themselves and their children, the study highlighted inadequate safety practices during pesticide use, exacerbated by a lack of formal training. Participants expressed concerns about the effects of pesticide exposure on themselves and their children, emphasizing the need for government-supported training on pesticide safety and use.

**Figure 2.**
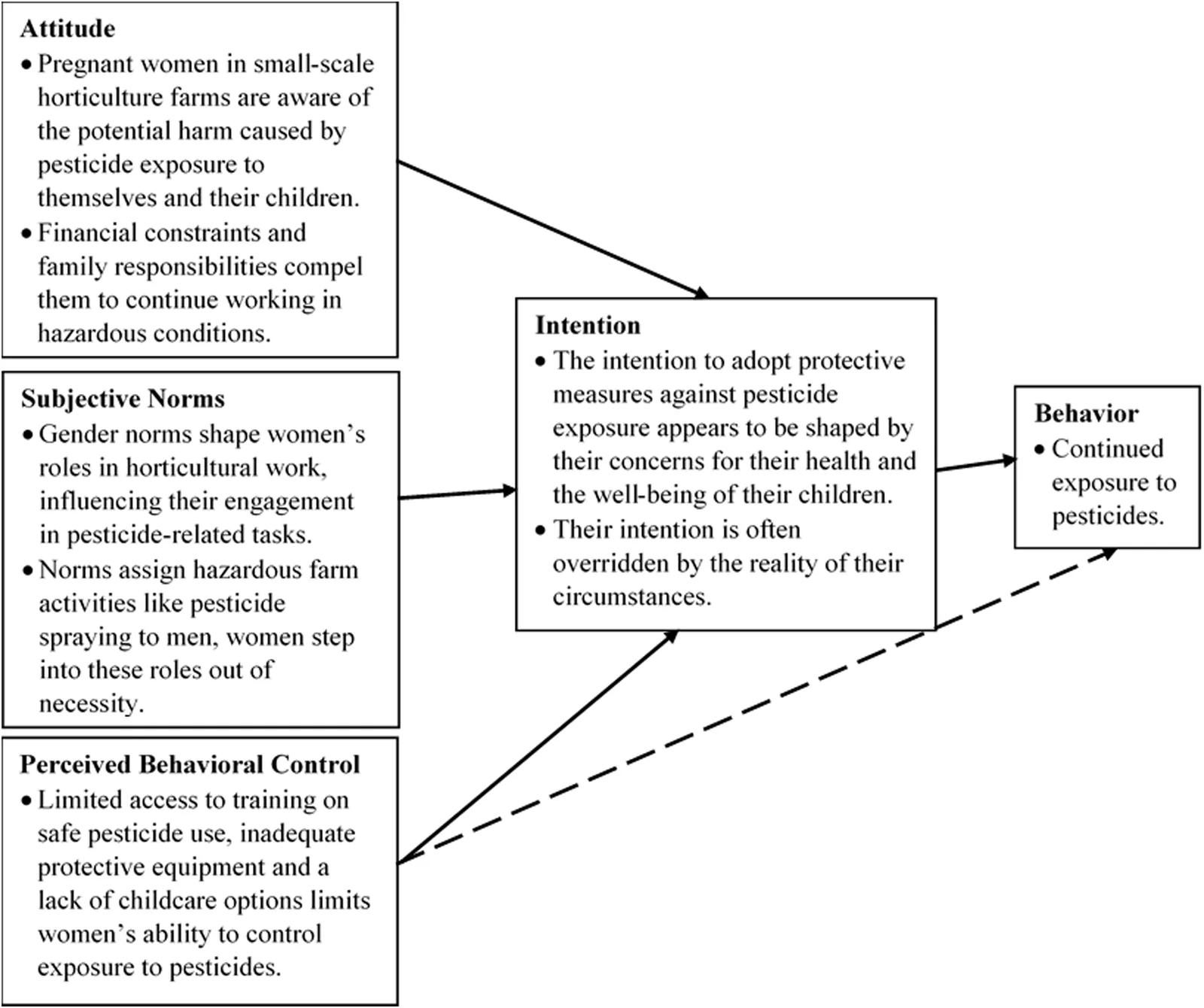
Dimensions of pesticide exposure among women and their children in small-scale horticulture farms under the theory of planned behavior.

The gendered division of labor in horticultural work reflects deep-rooted social norms, a pattern observed in multiple studies. Historically, men’s roles in agriculture were confined to physical labor while limiting women to tasks near the home (Hansen et al., 2015; Leipert & Reutter, 2005). That is why even women in horticulture today often perform tasks considered less hazardous and supportive in nature. Building on to this concept, Contzen and Forney explored gender roles in family farming and found that traditional divisions of labor align with longstanding gender norms that allocate primary production tasks like farm preparation and pesticide application to men and supportive roles like weeding and harvesting to women (Contzen & Forney, 2017). This dynamic also appears in Ghana, where national household survey data show that women are more engaged in cultivating crops for sustaining the family, while men focus on crops for commercial sale (Doss, 2002). This socially shaped division of labor tends to put women’s safety on the frontline; that is why even when women sometimes cross into tasks typically reserved for men, like pesticide spraying, they are considered to be occasional roles.

The tendency of women getting involved in tasks traditionally done by men varies across countries. Almost half of the women engaged in small-scale farming in KwaZulu-Natal, South Africa, indicated that they were primarily responsible for pesticide spraying (Naidoo et al., 2010). This might be indicative of the changes in the gender dynamics within agriculture as women are taking up roles traditionally dominated by men when required (Pfammatter & Jongerden, 2023). Nevertheless, it is not a universal trend. In Kenya, women’s participation in pesticide spraying remains limited as it is primarily carried out by men (Tsimbiri et al., 2015). Conversely, a review study about “women in the growth of horticulture” in India points out that women are often excluded from pesticide application, attributed to their lack of skills (Srivastava et al., 2020). Hence, even though women are getting involved in manly roles, the extent of their involvement is still shaped by the gender norms and labor needs.

The experiences of pregnant women working in horticulture exemplify the broader challenges women face across the agricultural sector. These challenges stem in part from financial dependency and inadequate support structures that often leave women with limited options. As Shekhar and Singh note, despite existing policy initiatives, women farmers remain significantly under-supported, facing barriers that limit their access to crucial benefits such as adequate rest during pregnancy and after childbirth (Shekhar & Singh, 2023). For many women, including those in this study, economic pressures and family responsibilities necessitate continued labor, often at the expense of their health. Consistent with these findings, Asmare et al. highlight that women on smallholder farms frequently undertake hazardous tasks due to economic constraints, with their health needs often overlooked as a result (Atinkut Asmare et al., 2022). A multi-country value chain study from Bangladesh, the Philippines, Benin, and Malawi similarly underscores how financial hardship forces women into labor-intensive roles, limiting their capacity to prioritize their health (Quisumbing et al., 2021). There is, therefore, an urgent need for policies that not only provide economic support to women farmers but also safeguard their health during pregnancy and postpartum periods.

Women working in horticulture face significant risk from pesticide exposure, and because they continue working through pregnancy, their unborn children are also at risk. The impact of exposure to pesticides during pregnancy has been adequately documented. There are reports suggesting adverse pregnancy outcomes like preterm birth (Jaacks et al., 2019; Lin et al., 2023), small for gestation age, and low birth weight (Jaacks et al., 2019) among exposed mothers. On the other hand, children experience double exposure: first in utero and then in the agricultural environment (Lu et al., 2000). There are reports on cognitive and motor development impairment (Bliznashka et al., 2023; Rowe et al., 2016), neural tube defects (Felisbino et al., 2024), and other neurodevelopment disorders (Gunier et al., 2017; Shelton et al., 2014) in children exposed to pesticides in early stages of life. As echoed by participants in this study, this high risk of exposure is often due to a lack of basic knowledge about pesticide handling and an unawareness of necessary safety precautions. Studies from South Africa (Naidoo et al., 2010), Nepal (Atreya, 2007), and India (Mancini et al., 2009) confirm that women in small-scale agriculture, who often have limited access to pesticide training, follow few safety practices. In addition, the risk is aggravated by a limited understanding of pesticide hazards and an inability to interpret label warnings, as reported in a qualitative policy study in South Africa by Rother et al. (2008). Environmental exposure happens in children as a result of limited access to affordable childcare. Many women in this study reported bringing their children to the fields, sometimes even while pesticides are actively being sprayed in neighboring farms or soon after, unknowingly putting themselves and their children at risk. This has also been reported by female cotton growers in India (Mancini et al., 2005) and a review study on female horticulture workers in Tanzania (Mrema et al., 2017). As reported by participants in this study, sometimes children visit sprayed farms themselves to play, which has also been reported from a study in Ecuador (Handal et al., 2007). Thus, there is a need for stronger protective measures for both mothers and children living and working in farming communities. There are documented interventions which have been proven to reduce the risk of pesticide poisoning in women and children. Some proposed interventions include selection and approval of only few safe pesticides for use (Damalas & Koutroubas, 2016; Jepson et al., 2020) and targeted educational programs like the use of personal protective equipment (PPE) (Damalas & Koutroubas, 2016; Perry & Layde, 2003), training of farm worker families (Quandt et al., 2013), home-based health education intervention by healthcare workers (Salvatore et al., 2015), or training in indigenous language. All these interventions showed significant reduction in pesticide exposure to the targeted groups. However, they were more effective when farm workers were provided with some basic equipment like PPE for compliance with recommended protective practices instead of only providing theoretical instruction.

### Limitations and Future Research Directions

This study has some limitations that future research should address. The data were collected from specific rural areas, which may limit how well the findings apply to other regions with different social and agricultural practices. Also, while the study highlights the role of gender norms and household income in shaping behaviors, it does not deeply explore how factors like age and education influence women’s attitudes, intentions, and behaviors. Comparative studies across different geographic and cultural contexts could provide broader insights into how local norms and resources impact behavior. Interventions involving the entire household or community, including men, could be evaluated to understand their effectiveness in changing norms and improving safety practices in small-scale horticulture. In addition, it might be an information bias related to the researchers in this study, as they were aware of many studies about the adverse health effects related to the pesticides. However, this bias might be minor, as the adverse health effects were not the main topic of the study.

## Conclusions

This study highlights the complex interplay between gendered labor roles, poverty, and pesticide exposure in small-scale horticulture, shedding light on the occupational health challenges faced by rural women. The findings reveal that traditional gender roles dictate labor divisions, with men primarily handling physically demanding tasks such as pesticide spraying, but economic hardships often compel women to engage in hazardous activities, including pesticide use, even during pregnancy and postpartum periods. This is especially true for poorer women or those without a husband. The lack of adequate training on pesticide safety further exacerbates health risks for both women and their children. Gender-sensitive occupational health training, improved access to protective equipment, and reducing the economic pressures faced by women working in small-scale horticulture might minimize the risk.
